# Synthesis, crystal structure and Hirshfeld surface analysis of 1-(3,4-di­chloro­phen­yl)-4,4,6-trimethyl-3,4-di­hydro­pyrimidine-2(1*H*)-thione

**DOI:** 10.1107/S2056989026007231

**Published:** 2026-07-16

**Authors:** Sharatha Kumar, Tuncer Hökelek

**Affiliations:** a Department of Chemistry Yenepoya Institute of Arts Science Commerce and Management Mangaluru Yenepoya University (Deemed to be University) 575013 Karnataka India; b Hacettepe University, Department of Physics, 06800 Beytepe-Ankara, Türkiye; Universidad de la Repüblica, Uruguay

**Keywords:** pyrimidine, 3,4-di­chloro­phen­yl, crystal structure

## Abstract

The title compound, C_13_H_14_Cl_2_N_2_S, consists of di­chloro­phenyl and di­hydro­pyrimidine­thione rings, where the pyrimidine ring is in a flattened-boat conformation. In the crystal, N—H⋯S hydrogen bonds link the mol­ecules, enclosing *R*^2^_2_(8) ring motifs, into centrosymmetric dimers. Neither π–π stacking nor C—H⋯π(ring) inter­actions are observed. Hirshfeld surface analysis revealed that the most important contributions for crystal packing are from H⋯H (40.8%), H⋯Cl/Cl⋯H (28.7%) and H⋯S/S⋯H (15.5%) inter­actions. The volume of the crystal voids and the percentage of free space were calculated to be 135.01 Å^3^ and 17.99%, showing the crystal packing is not compact. Computational methods indicated an N—H⋯S hydrogen-bonding energy of −58.2 kJ mol^−1^. Evaluations of the electrostatic, dispersion and total energy frameworks indicate that the crystal cohesion is dominated by electrostatic energy contributions.

## Abstract 2

The title compound consists of di­chloro­phenyl and di­hydro­pyrimidine­thione rings, where the pyrimidine ring is in flattened-boat conformation. In the crystal, N—H⋯S hydrogen bonds link the mol­ecules, enclosing *R*^2^_2_(8) ring motifs, into centrosymmetric dimers.

## 1. Chemical context

Heterocyclic compounds containing pyrimidine and di­hydro­pyrimidine frameworks have attracted sustained inter­est because of their wide range of biological and pharmacological activities, including anti­bacterial, anti­fungal, anti­tubercular and anti-inflammatory properties (Kappe, 2000 ▸; Leite *et al.*, 2006 ▸; Sriram *et al.*, 2006 ▸). In particular, Biginelli-type di­hydro­pyrimidine derivatives are recognized as important structural motifs in medicinal chemistry and synthetic organic chemistry owing to their diverse therapeutic potentials and convenient synthetic accessibilities.
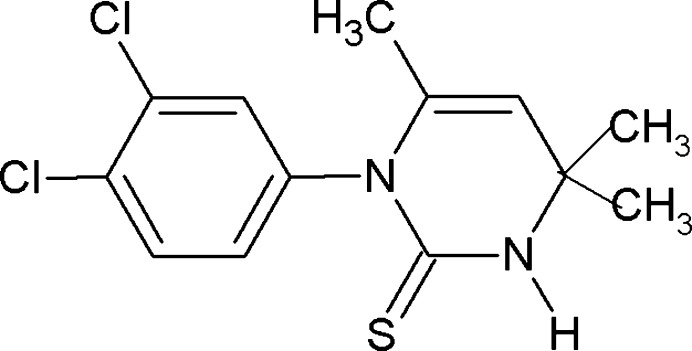


Continuing our inter­est in the syntheses and structural characterizations of biologically significant heterocyclic compounds (Alam *et al.*, 2005 ▸), the title compound was prepared and investigated. Herein, we report its mol­ecular and crystal structures together with the results of Hirshfeld surface (HS), crystal void analyses and inter­action energy calculations and energy frameworks of the title compound, (I).

## 2. Structural commentary

**Figure 1:**
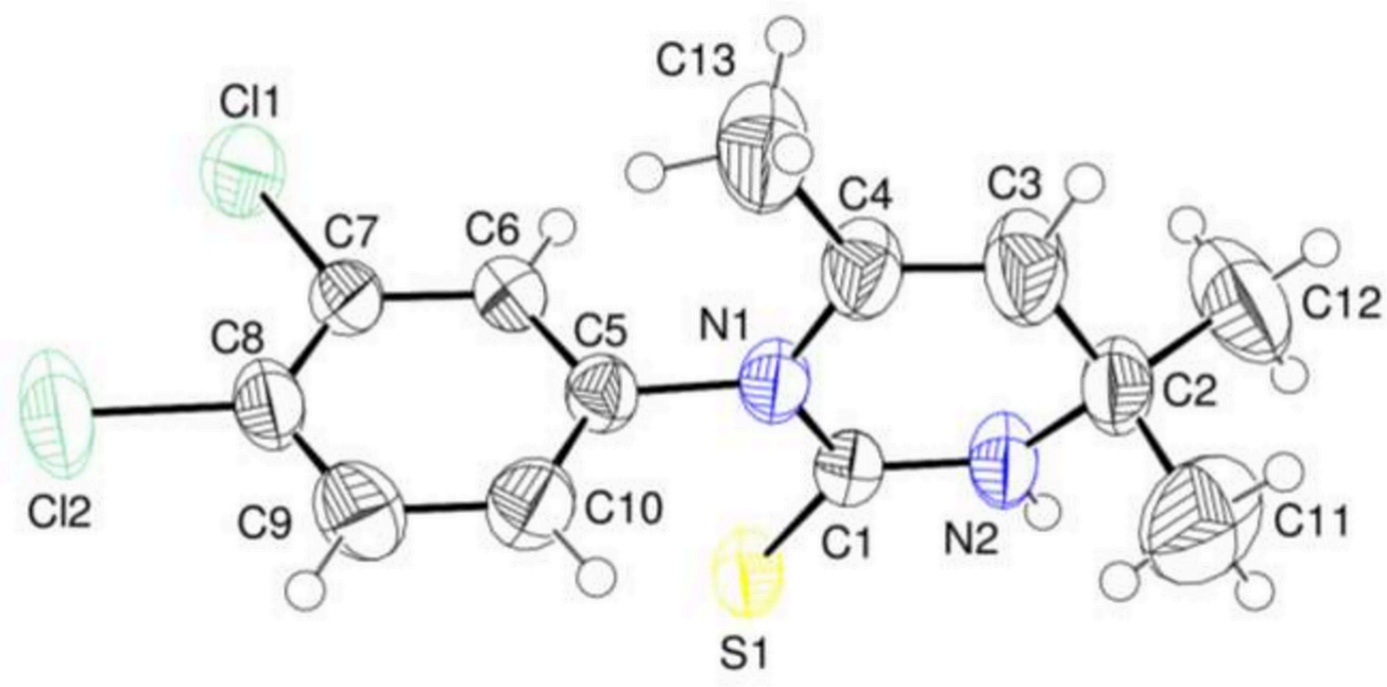
The asymmetric unit with the atom-numbering scheme and 50% probability ellipsoids.

**Figure 2:**
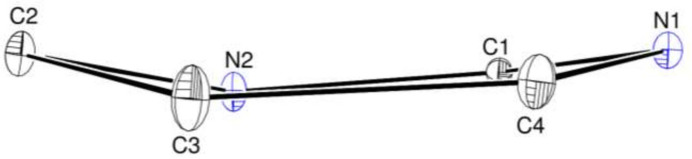
Conformation of the pyrimidine ring.

The title compound, (I), contains a planar phenyl ring and a six-membered nitro­gen-containing heterocyclic di­hydro­pyrimidine­thione ring (Fig. 1 ▸). The 3,4-di­chloro­phenyl, dimethyl and methyl substituents are bonded to the 3,4-di­hydro­pyrimidine-2(*1H*)-thione ring at the N1, C2 and C4 positions, respectively. The pyrimidine, *A* (N1/N2/C1–C4), ring is in flattened-boat conformation (Fig. 2 ▸) with puckering parameters (Cremer & Pople, 1975 ▸) *Q*_T_ = 0.184 (3) Å, θ = 70.8 (9)° and φ = 174.4 (11)°. It is reported to be planar in 4,4,6-trimethyl-1-phenyl-3,4-di­hydro-pyrimidine-2(1*H*)-thione [(II); Yamin *et al.*, 2005 ▸], N*-*benzoyl-*N*′-phenyl­thio­urea [(III); Yamin & Yusof, 2003 ▸] and 1-ethyl-5-(4-meth­oxy­benzo­yl)-4-(4-meth­oxy­phen­yl)pyrimidine-2(*1H*)-thione [(IV); Özçelik *et al.*, 2004 ▸]. The S1=C1 [1.692 (2) Å], C1—N1 [1.361 (3) Å] and C1—N2 [1.323 (3) Å] bond lengths are comparable with the corresponding values in compounds II—IV. Other bond lengths are in normal ranges (Allen *et al.*, 1987 ▸).

## 3. Supra­molecular features, Hirshfeld surface analysis, void analysis, inter­action energies and energy frameworks

**Figure 3:**
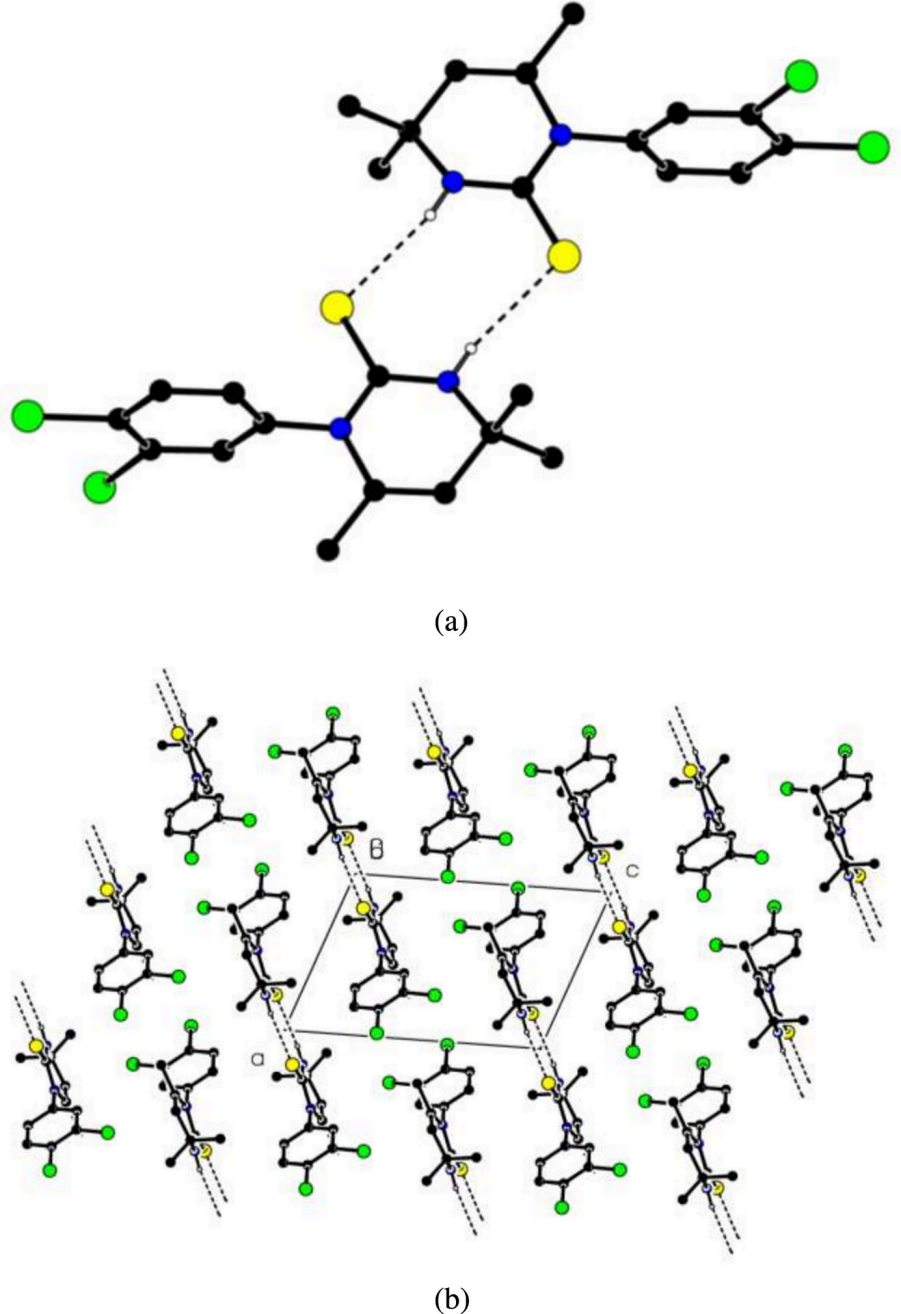
(*a*) The dimer formation through N—H⋯S hydrogen bonds (shown as dashed lines) with an 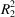
(8) ring motif. (*b*) A partial packing diagram viewed down the *b*-axis direction showing the supra­molecular dimer formation. The N—H⋯S hydrogen bonds are shown as dashed lines.

In the crystal, N—H⋯S hydrogen bonds (Table 1 ▸) link the mol­ecules, enclosing 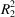
(8) ring motifs (Etter *et al.*, 1990 ▸), into centrosymmetric dimers (Fig. 3 ▸*a*). The supra­molecular dimer formation through the N—H⋯S hydrogen bonds is shown in Fig. 3 ▸*b*. Neither π–π stacking nor C—H⋯π(ring) inter­actions are observed.

**Figure 4:**
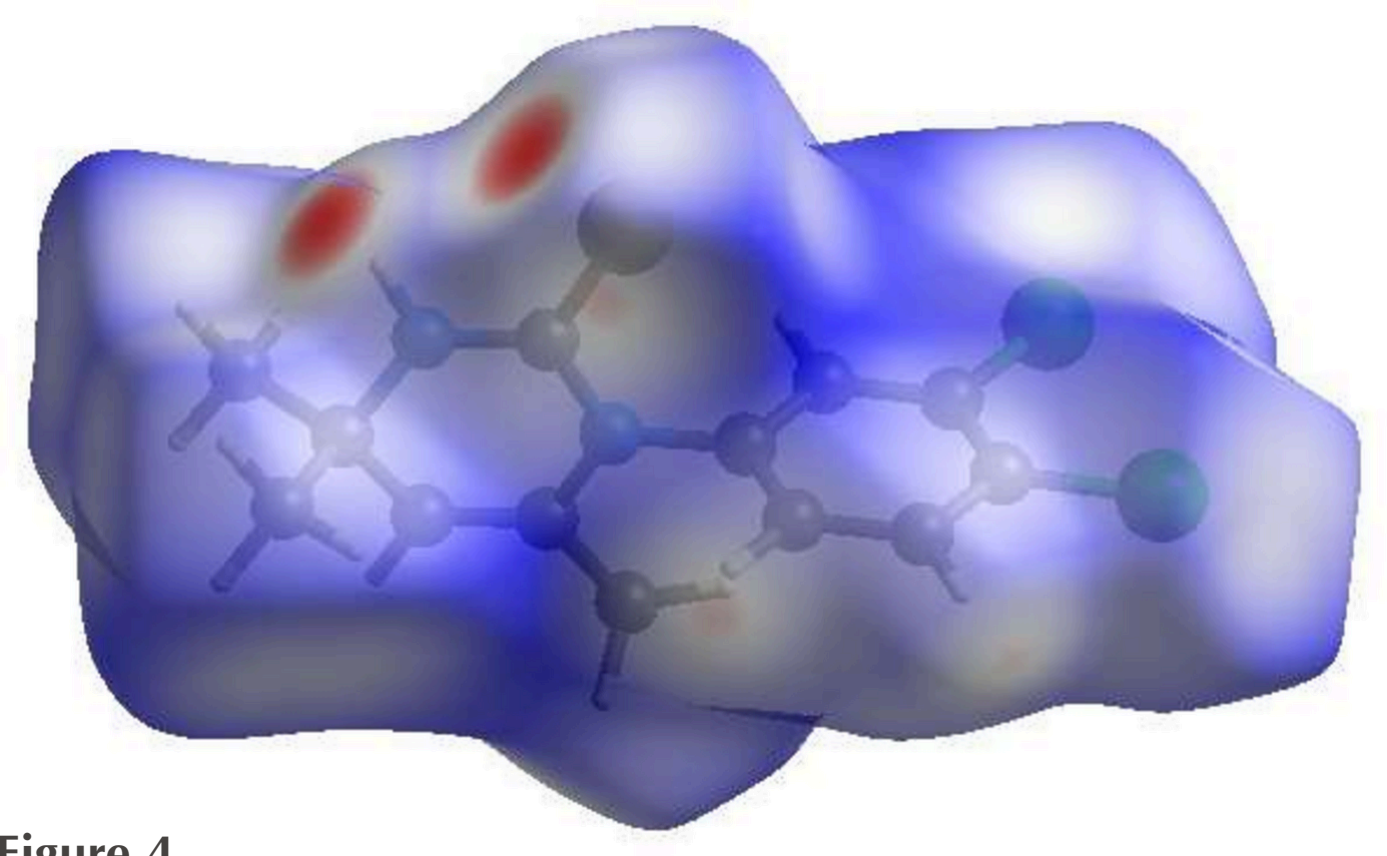
View of the three-dimensional Hirshfeld surface plotted over *d*_norm_ in the range −0.3452 to 1.4957 a.u.

**Figure 5:**
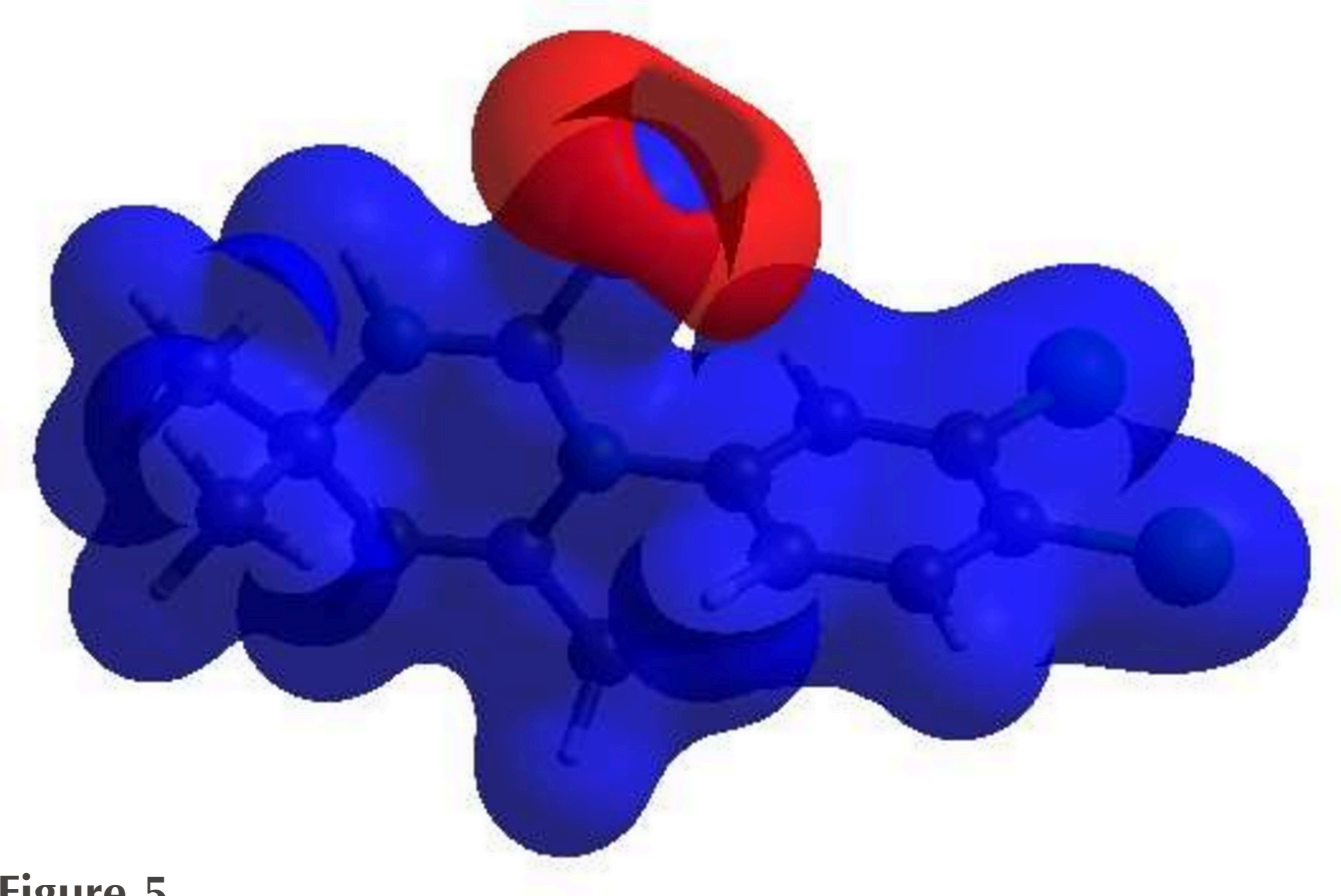
View of the three-dimensional Hirshfeld surface of the title compound plotted over electrostatic potential in the range −0.0500 to 0.0500 a.u. using the STO-3 G basis set at the Hartree–Fock level of theory. Hydrogen-bond donors and acceptors are shown as blue and red regions around the atoms, corresponding to positive and negative potentials, respectively.

The inter­molecular inter­actions in the crystal were visualized by carrying out a Hirshfeld surface (HS) analysis using *CrystalExplorer* 17.5 (Spackman *et al.*, 2021 ▸). Fig. 4 ▸ shows the Hirshfeld surface with several neighbouring mol­ecules in the crystal. The white surface indicates contacts with distances equal to the sum of van der Waals radii, and the red and blue colours indicate distances shorter (in close contact) or longer (distant contacts) than the sum of the van der Waals radii, respectively. The red spots indicate their roles as the respective donors and/or acceptors atoms in hydrogen bonding, as discussed above; they also appear as the blue and red regions corresponding to positive and negative potentials on the HS mapped over electrostatic potential as shown in Fig. 5 ▸. The blue and red regions indicate positive (hydrogen-bond donors) and negative (hydrogen-bond acceptors) electrostatic potentials.

**Figure 6:**
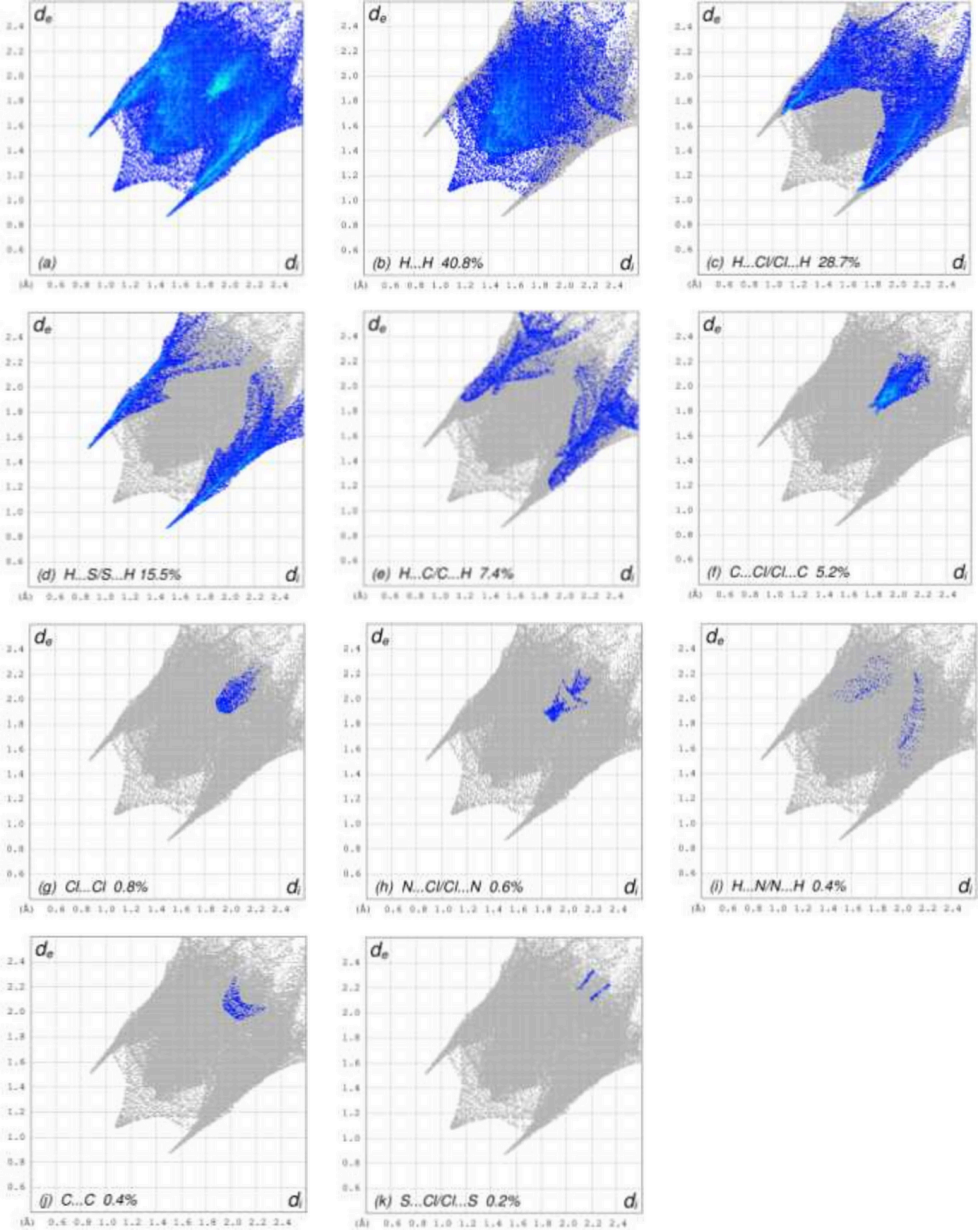
The two-dimensional fingerprint plots for title mol­ecule, showing (*a*) all inter­actions, and delineated into (*b*) H⋯H, (*c*) H⋯Cl/Cl⋯H, (*d*) H⋯S/S⋯H, (*e*) H⋯C/C⋯H, (*f*) C⋯Cl/Cl⋯C, (*g*) Cl⋯Cl, (*h*) N⋯Cl/Cl⋯N, (i) H⋯N/N⋯H, (*j*) C⋯C and (*k*) S⋯Cl/Cl⋯S inter­actions. The *d*_i_ and *d*_e_ values are the closest inter­nal and external distances (in Å) from given points on the Hirshfeld surface.

The overall two-dimensional fingerprint plot is shown in Fig. 6 ▸*a* and those delineated into H⋯H, H⋯Cl/Cl⋯H, H⋯S/S⋯H, H⋯C/C ⋯ H, C⋯Cl/Cl⋯C, Cl⋯Cl, N⋯Cl/Cl⋯N, H⋯N/N⋯H, C⋯C and S⋯Cl/Cl⋯S inter­actions are illustrated in Fig. 6 ▸(*b*)–(*k*), respectively. According to the two-dimensional fingerprint plots the H⋯H, H⋯Cl/Cl⋯H, and H⋯S/S⋯H contacts make the most significant contributions to the HS, at 40.8%, 28.7% and 15.5%, respectively (Fig. 6 ▸).

**Figure 7:**
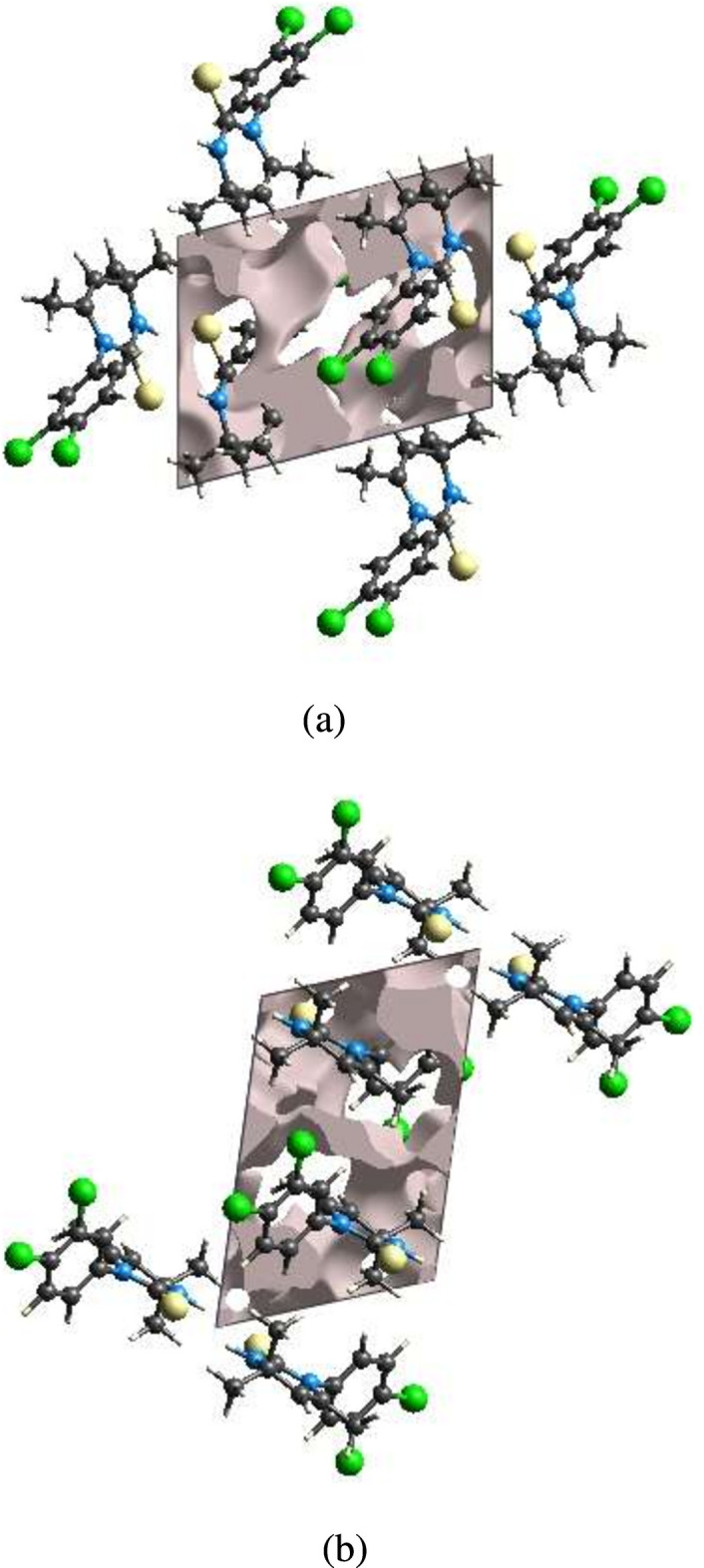
Crystal voids viewed down the (*a*) *a*-axis and (*b*) *b*-axis directions.

The strength of the crystal packing depends on the tight packing of the mol­ecules, which results in insignificant voids. To check the strength of the crystal, a void analysis was performed. The volume of the crystal voids (Fig. 7 ▸*a* and *b*) and the percentage of free space in the unit cell were calculated to be 135.01 Å^3^ and 17.99%, respectively. Thus, the crystal packing appears to be not compact.

The inter­molecular inter­action energies are calculated using the CE–B3LYP/6–31G(d,p) energy model available in *CrystalExplorer* 17.5 (Spackman *et al.*, 2021 ▸), where a cluster of mol­ecules is generated by applying crystallographic symmetry operations with respect to a selected central mol­ecule within the radius of 3.8 Å by default. The total inter­molecular energy (*E*_tot_) is the sum of electrostatic (*E*_ele_), polarization (*E*_pol_), dispersion (*E*_dis_) and exchange-repulsion (*E*_rep_) energies (Turner *et al.*, 2015 ▸) with scale factors of 1.057, 0.740, 0.871 and 0.618, respectively (Mackenzie *et al.*, 2017 ▸). Hydrogen-bonding inter­action energies (in kJ mol^−1^) were calculated to be −80.2 (*E*_ele_), −20.0 (*E*_pol_), −22.8 (*E*_dis_), 70.4 (*E*_rep_) and −58.2 (*E*_tot_) for N2—H2*N*⋯S1.

**Figure 8:**
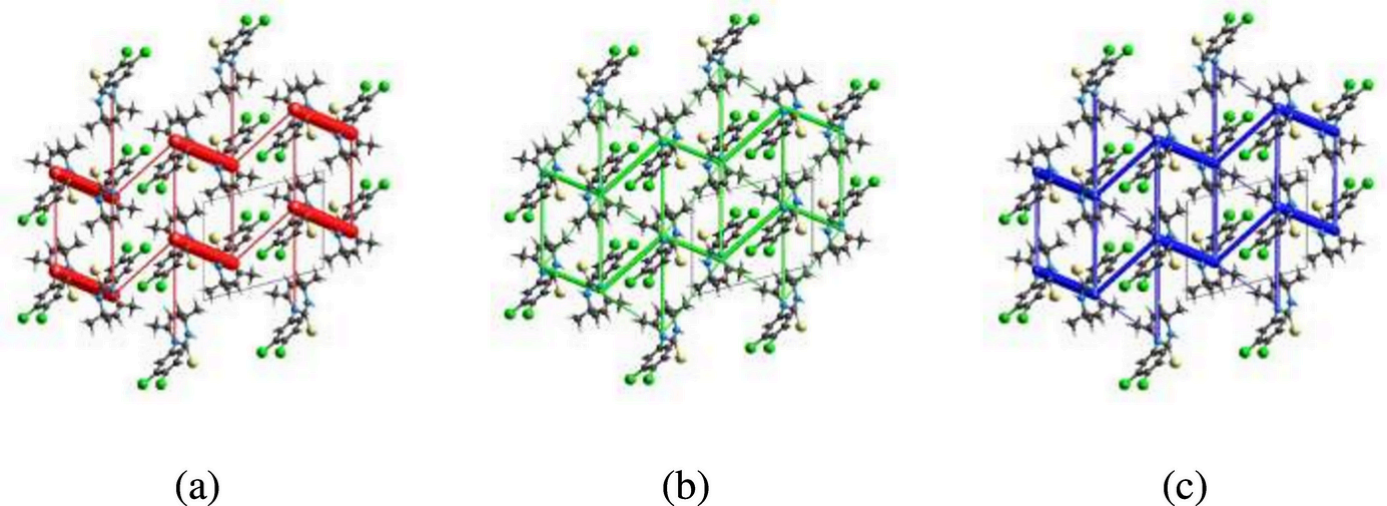
The energy frameworks for a cluster of mol­ecules of the title compound viewed down the *a*-axis showing the (*a*) electrostatic energy, (*b*) dispersion energy and (*c*) total energy diagrams. The cylindrical radius is proportional to the relative strength of the corresponding energies and they were adjusted to the same scale factor of 80 with cut-off value of 5 kJ mol^−1^ within 2 × 2 × 2 unit cells.

Energy frameworks combine the calculation of inter­molecular inter­action energies with a graphical representation of their magnitudes, in which they were constructed for *E*_ele_ (red cylinders), *E*_dis_ (green cylinders) and *E*_tot_ (blue cylinders) (Fig. 8 ▸*a*, *b* and *c*). The evaluations of the electrostatic, dispersion and total energy frameworks indicate that the stabilization is dominated *via* the electrostatic energy contributions in the crystal structure of the title compound.

## 4. Database survey

A search of the Cambridge Structural Database (CSD, Version 6.00, updated May 2025; Groom *et al.*, 2016 ▸) revealed six closely related structures of 1-aryl-4,4,6-trimethyl-3,4-di­hydro­pyrimidine-2(1*H*)-thione derivative, *viz.*: 1-(4-fluoro­phen­yl)-4,4,6-trimethyl-3,4-di­hydro­pyrimidine-2(1*H*)-thione, C_13_H_15_FN_2_S (CSD refcode ASEHIR; Kadir *et al.*, 2016 ▸), 1-(3-chloro­phen­yl)-4,4,6-trimethyl-3,4-di­hydro­pyrimidine-2(1*H*)-thione, C_13_H_15_ClN_2_S (CSD refcode IJUGEA; Yamin & Salem, 2011*a* ▸), 1-(3-fluoro­phen­yl)-4,4,6-trimethyl-3,4-di­hydro­pyrim­idine-2(1*H*)-thione, C_13_H_15_FN_2_S (CSD refcode EVEWIM; Yamin *et al.*, 2011*b* ▸), 4,4,6-trimethyl-1-phenyl-3,4-di­hydro­pyrimidine-2(1*H*)-thione, C_13_H_16_N_2_S (Yamin *et al.*, 2005 ▸), 1-(4-chloro­phen­yl)-4,4,6-trimethyl-3,4-di­hydro­pyrimidine-2(1*H*)-thione, C_13_H_15_ClN_2_S (CSD refcode DUNZIW; Saeed & Bolte, 2010*a* ▸) and 4,4,6-trimethyl-1-(3-methyl­phen­yl)-3,4-di­hydro­pyrimidine-2(1*H*)-thione, C_14_H_18_N_2_S (CSD refcode PUJYID; Saeed *et al.*, 2010*b* ▸). The title compound differs from these reported structures by the presence of chlorine substituents at both the 3- and 4- positions of the phenyl ring. Similar to several related derivatives, the crystal packing is consolidated by N—H⋯S hydrogen bonds, forming centrosymmetric dimers.

## 5. Synthesis and crystallization

3,4-Di­chloro aniline (0.16 g, 1.0 mmol) was added portion-wise to a stirred solution of potassium thio­cyanate (0.09 g, 1.0 mmol) in acetone containing one drop of H_2_SO_4_ at room temperature. The reaction mixture was then heated at 232–333 K for 3 h while being monitored using thin-layer chromatography. After the reaction was complete, the content was cooled to room temperature, and then poured into ice–water. The resulting precipitate was filtered, dried and recrystallized at room temperature from ethanol solution by gradual evaporation. Colourless crystals suitable for X-ray analysis were obtained by slow evaporation of aceto­nitrile. Colourless, yield 86%, m.p. 468 K, IR (KBr) cm^−1^: 3285 (N—H str.), 1530 (C=S str.), 3176 (Ar. C—H str.).

## 6. Refinement

Crystal data, data collection and structure refinement details are summarized in Table 2 ▸. The NH hydrogen atoms were located from difference-Fourier maps and refined isotropically. The C-bound hydrogen-atom positions were calculated geometrically at distances of 0.93 Å (for aromatic CH) and 0.96 Å (for methyl CH) and refined using a riding model by applying the constraint *U*_iso_(H) = *k* × U_eq_ (C), where *k* = 1.5 for methyl hydrogens and *k* = 1.2 for the other H atoms.

## Supplementary Material

Crystal structure: contains datablock(s) I, global. DOI: 10.1107/S2056989026007231/ny2020sup1.cif

Structure factors: contains datablock(s) I. DOI: 10.1107/S2056989026007231/ny2020Isup2.hkl

printcif, checkcif. DOI: 10.1107/S2056989026007231/ny2020sup3.pdf

Supporting information file. DOI: 10.1107/S2056989026007231/ny2020sup4.pdf

check cif. DOI: 10.1107/S2056989026007231/ny2020sup5.pdf

Supporting information file. DOI: 10.1107/S2056989026007231/ny2020sup6.pdf

CCDC reference: 1816810

Additional supporting information:  crystallographic information; 3D view; checkCIF report

## Figures and Tables

**Table 1:** Hydrogen-bond geometry (Å, °)

*D*—H⋯*A*	*D*—H	H⋯*A*	*D*⋯*A*	*D*—H⋯*A*
N2—H2*N*⋯S1^i^	0.85 (2)	2.55 (2)	3.375 (2)	163 (3)

Symmetry code: (i) 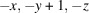.

**Table 2:** Experimental details

Crystal data	
Chemical formula	C_13_H_14_Cl_2_N_2_S
*M*_r_	301.22
Crystal system, space group	Triclinic, *P* 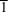
Temperature (K)	293
*a*, *b*, *c* (Å)	7.916 (1), 8.927 (1), 11.759 (2)
α, β, γ (°)	99.93 (1), 107.18 (1), 102.32 (1)
*V* (Å^3^)	750.52 (19)
*Z*	2
Radiation type	Mo *K*α
μ (mm^−1^)	0.56
Crystal size (mm)	0.50 × 0.36 × 0.34

Data collection	
Diffractometer	Oxford Diffraction Xcalibur with Sapphire CCD
Absorption correction	Multi-scan (*CrysAlis RED*; Oxford Diffraction, 2006 ▸
*T*_min_, *T*_max_	0.769, 0.834
No. of measured, independent and observed [*I* > 2σ(*I*)] reflections	5185, 3026, 2502
*R*_int_	0.014
(sin θ/λ)_max_ (Å^−1^)	0.625

Refinement	
*R*[*F*^2^ > 2σ(*F*^2^)], *wR*(*F*^2^), *S*	0.047, 0.131, 1.09
No. of reflections	3026
No. of parameters	166
No. of restraints	1
H-atom treatment	H atoms treated by a mixture of independent and constrained refinement
Δρ_max_, Δρ_min_ (e Å^−3^)	0.34, −0.36

Computer programs: *CrysAlis CCD* and *CrysAlis RED* (Oxford Diffraction, 2006 ▸), *SHELXT2014/5* (Sheldrick, 2015*a* ▸), *SHELXL2018/3* (Sheldrick, 2015*b* ▸), *ORTEP-3 for Windows* and *WinGX* publication routines (Farrugia, 2012 ▸) and *PLATON* (Spek, 2020 ▸).

## supplementary crystallographic information

### 1-(3,4-Dichlorophenyl)-4,4,6-trimethyl-3,4-dihydropyrimidine-2(1H)-thione Crystal data

**Table:**

C_13_H_14_Cl_2_N_2_S	*Z* = 2
*M**_r_* = 301.22	*F*(000) = 312
Triclinic, *P*1	*D*_x_ = 1.333 Mg m^−^^3^
*a* = 7.916 (1) Å	Mo *K*α radiation, λ = 0.71073 Å
*b* = 8.927 (1) Å	Cell parameters from 2526 reflections
*c* = 11.759 (2) Å	θ = 2.7–27.9°
α = 99.93 (1)°	µ = 0.56 mm^−^^1^
β = 107.18 (1)°	*T* = 293 K
γ = 102.32 (1)°	Prism, colourless
*V* = 750.52 (19) Å^3^	0.50 × 0.36 × 0.34 mm

### 1-(3,4-Dichlorophenyl)-4,4,6-trimethyl-3,4-dihydropyrimidine-2(1H)-thione Data collection

**Table:**

Oxford Diffraction Xcalibur with Sapphire CCD diffractometer	2502 reflections with *I* > 2σ(*I*)
Rotation method data acquisition using ω scans.	*R*_int_ = 0.014
Absorption correction: multi-scan (CrysAlis RED; Oxford Diffraction, 2006	θ_max_ = 26.4°, θ_min_ = 2.7°
*T*_min_ = 0.769, *T*_max_ = 0.834	*h* = −9→9
5185 measured reflections	*k* = −10→11
3026 independent reflections	*l* = −14→14

### 1-(3,4-Dichlorophenyl)-4,4,6-trimethyl-3,4-dihydropyrimidine-2(1H)-thione Refinement

**Table:**

Refinement on *F*^2^	1 restraint
Least-squares matrix: full	Hydrogen site location: mixed
*R*[*F*^2^ > 2σ(*F*^2^)] = 0.047	H atoms treated by a mixture of independent and constrained refinement
*wR*(*F*^2^) = 0.131	*w* = 1/[σ^2^(*F*_o_^2^) + (0.0531*P*)^2^ + 0.5196*P*] where *P* = (*F*_o_^2^ + 2*F*_c_^2^)/3
*S* = 1.09	(Δ/σ)_max_ < 0.001
3026 reflections	Δρ_max_ = 0.34 e Å^−^^3^
166 parameters	Δρ_min_ = −0.36 e Å^−^^3^

### 1-(3,4-Dichlorophenyl)-4,4,6-trimethyl-3,4-dihydropyrimidine-2(1H)-thione Special details

**Table:**

Geometry. All esds (except the esd in the dihedral angle between two l.s. planes) are estimated using the full covariance matrix. The cell esds are taken into account individually in the estimation of esds in distances, angles and torsion angles; correlations between esds in cell parameters are only used when they are defined by crystal symmetry. An approximate (isotropic) treatment of cell esds is used for estimating esds involving l.s. planes.

### 1-(3,4-Dichlorophenyl)-4,4,6-trimethyl-3,4-dihydropyrimidine-2(1H)-thione Fractional atomic coordinates and isotropic or equivalent isotropic displacement parameters (Å^2^)

**Table:**

	*x*	*y*	*z*	*U*_iso_*/*U*_eq_	
C1	0.3026 (3)	0.5937 (3)	0.1548 (2)	0.0422 (5)	
C2	0.2695 (4)	0.8680 (3)	0.1621 (3)	0.0603 (7)	
C3	0.4444 (5)	0.9152 (4)	0.2678 (4)	0.0816 (11)	
H3	0.485846	1.019411	0.314955	0.098*	
C4	0.5456 (4)	0.8184 (3)	0.2995 (3)	0.0627 (8)	
C5	0.5946 (3)	0.5528 (3)	0.2628 (2)	0.0434 (5)	
C6	0.5958 (3)	0.4814 (3)	0.3573 (2)	0.0465 (6)	
H6	0.516423	0.494028	0.400471	0.056*	
C7	0.7150 (4)	0.3909 (3)	0.3879 (2)	0.0480 (6)	
C8	0.8296 (4)	0.3696 (3)	0.3222 (3)	0.0540 (6)	
C9	0.8260 (4)	0.4402 (4)	0.2263 (3)	0.0649 (8)	
H9	0.902697	0.425268	0.181383	0.078*	
C10	0.7088 (4)	0.5327 (4)	0.1971 (3)	0.0574 (7)	
H10	0.707063	0.581304	0.133010	0.069*	
C11	0.2915 (7)	0.9217 (5)	0.0513 (4)	0.1110 (16)	
H11A	0.333986	1.035250	0.071888	0.166*	
H11B	0.379489	0.878031	0.026262	0.166*	
H11C	0.174966	0.885896	−0.014726	0.166*	
C12	0.1231 (6)	0.9308 (5)	0.1992 (5)	0.1088 (15)	
H12A	0.162291	1.044564	0.221850	0.163*	
H12B	0.008837	0.894392	0.131086	0.163*	
H12C	0.106251	0.892958	0.267781	0.163*	
C13	0.7245 (5)	0.8681 (4)	0.4024 (4)	0.0966 (14)	
H13A	0.773023	0.778897	0.408496	0.145*	
H13B	0.809630	0.949830	0.387448	0.145*	
H13C	0.706552	0.907897	0.477922	0.145*	
N1	0.4778 (3)	0.6555 (2)	0.23637 (19)	0.0465 (5)	
N2	0.2043 (3)	0.6938 (3)	0.1281 (2)	0.0554 (6)	
Cl1	0.71964 (14)	0.30829 (10)	0.51126 (7)	0.0761 (3)	
Cl2	0.98030 (13)	0.25664 (12)	0.35758 (10)	0.0907 (3)	
S1	0.21347 (9)	0.39621 (7)	0.09242 (7)	0.0518 (2)	
H2N	0.094 (3)	0.652 (3)	0.078 (2)	0.062*	

### 1-(3,4-Dichlorophenyl)-4,4,6-trimethyl-3,4-dihydropyrimidine-2(1H)-thione Atomic displacement parameters (Å^2^)

**Table:**

	*U*^11^	*U*^22^	*U*^33^	*U*^12^	*U*^13^	*U*^23^
C1	0.0414 (12)	0.0430 (12)	0.0441 (12)	0.0150 (10)	0.0137 (10)	0.0139 (10)
C2	0.0642 (17)	0.0437 (14)	0.0679 (18)	0.0224 (13)	0.0089 (14)	0.0166 (13)
C3	0.079 (2)	0.0437 (16)	0.095 (3)	0.0202 (15)	−0.0047 (19)	0.0087 (16)
C4	0.0581 (16)	0.0415 (14)	0.0708 (19)	0.0116 (12)	0.0006 (14)	0.0107 (13)
C5	0.0388 (12)	0.0433 (12)	0.0455 (13)	0.0140 (10)	0.0106 (10)	0.0082 (10)
C6	0.0527 (14)	0.0453 (13)	0.0455 (13)	0.0204 (11)	0.0185 (11)	0.0102 (10)
C7	0.0562 (15)	0.0394 (12)	0.0436 (13)	0.0176 (11)	0.0097 (11)	0.0068 (10)
C8	0.0468 (14)	0.0476 (14)	0.0623 (16)	0.0227 (11)	0.0094 (12)	0.0052 (12)
C9	0.0557 (17)	0.078 (2)	0.0727 (19)	0.0291 (15)	0.0333 (15)	0.0167 (16)
C10	0.0533 (15)	0.0718 (18)	0.0558 (16)	0.0226 (13)	0.0234 (13)	0.0240 (14)
C11	0.144 (4)	0.072 (2)	0.103 (3)	0.007 (3)	0.028 (3)	0.042 (2)
C12	0.090 (3)	0.083 (3)	0.138 (4)	0.046 (2)	0.020 (3)	−0.007 (3)
C13	0.075 (2)	0.0499 (17)	0.118 (3)	0.0162 (16)	−0.022 (2)	0.0000 (18)
N1	0.0443 (11)	0.0431 (11)	0.0505 (12)	0.0162 (9)	0.0104 (9)	0.0134 (9)
N2	0.0468 (12)	0.0428 (12)	0.0681 (15)	0.0166 (10)	0.0048 (11)	0.0137 (10)
Cl1	0.1138 (7)	0.0656 (5)	0.0650 (5)	0.0473 (5)	0.0301 (5)	0.0312 (4)
Cl2	0.0794 (6)	0.0843 (6)	0.1211 (8)	0.0566 (5)	0.0286 (5)	0.0269 (5)
S1	0.0471 (4)	0.0416 (3)	0.0619 (4)	0.0150 (3)	0.0103 (3)	0.0129 (3)

### 1-(3,4-Dichlorophenyl)-4,4,6-trimethyl-3,4-dihydropyrimidine-2(1H)-thione Geometric parameters (Å, º)

**Table:**

C1—N2	1.323 (3)	C7—Cl1	1.732 (3)
C1—N1	1.361 (3)	C8—C9	1.379 (4)
C1—S1	1.692 (2)	C8—Cl2	1.726 (3)
C2—N2	1.472 (3)	C9—C10	1.378 (4)
C2—C3	1.480 (4)	C9—H9	0.9300
C2—C11	1.507 (5)	C10—H10	0.9300
C2—C12	1.529 (5)	C11—H11A	0.9600
C3—C4	1.326 (4)	C11—H11B	0.9600
C3—H3	0.9300	C11—H11C	0.9600
C4—N1	1.419 (3)	C12—H12A	0.9600
C4—C13	1.484 (4)	C12—H12B	0.9600
C5—C6	1.371 (3)	C12—H12C	0.9600
C5—C10	1.372 (4)	C13—H13A	0.9600
C5—N1	1.443 (3)	C13—H13B	0.9600
C6—C7	1.378 (3)	C13—H13C	0.9600
C6—H6	0.9300	N2—H2N	0.851 (17)
C7—C8	1.376 (4)		
			
N2—C1—N1	117.4 (2)	C8—C9—H9	120.0
N2—C1—S1	121.53 (19)	C5—C10—C9	119.7 (3)
N1—C1—S1	121.06 (17)	C5—C10—H10	120.1
N2—C2—C3	107.2 (2)	C9—C10—H10	120.1
N2—C2—C11	108.3 (3)	C2—C11—H11A	109.5
C3—C2—C11	113.0 (3)	C2—C11—H11B	109.5
N2—C2—C12	107.8 (3)	H11A—C11—H11B	109.5
C3—C2—C12	110.5 (3)	C2—C11—H11C	109.5
C11—C2—C12	109.8 (3)	H11A—C11—H11C	109.5
C4—C3—C2	124.2 (3)	H11B—C11—H11C	109.5
C4—C3—H3	117.9	C2—C12—H12A	109.5
C2—C3—H3	117.9	C2—C12—H12B	109.5
C3—C4—N1	119.2 (3)	H12A—C12—H12B	109.5
C3—C4—C13	123.9 (3)	C2—C12—H12C	109.5
N1—C4—C13	116.9 (2)	H12A—C12—H12C	109.5
C6—C5—C10	120.6 (2)	H12B—C12—H12C	109.5
C6—C5—N1	119.4 (2)	C4—C13—H13A	109.5
C10—C5—N1	119.9 (2)	C4—C13—H13B	109.5
C5—C6—C7	119.8 (2)	H13A—C13—H13B	109.5
C5—C6—H6	120.1	C4—C13—H13C	109.5
C7—C6—H6	120.1	H13A—C13—H13C	109.5
C8—C7—C6	120.0 (2)	H13B—C13—H13C	109.5
C8—C7—Cl1	121.2 (2)	C1—N1—C4	121.1 (2)
C6—C7—Cl1	118.7 (2)	C1—N1—C5	119.6 (2)
C7—C8—C9	119.9 (2)	C4—N1—C5	119.2 (2)
C7—C8—Cl2	121.4 (2)	C1—N2—C2	127.5 (2)
C9—C8—Cl2	118.8 (2)	C1—N2—H2N	116 (2)
C10—C9—C8	120.0 (3)	C2—N2—H2N	116 (2)
C10—C9—H9	120.0		
			
N2—C2—C3—C4	17.7 (5)	N2—C1—N1—C4	6.2 (4)
C11—C2—C3—C4	−101.6 (5)	S1—C1—N1—C4	−172.6 (2)
C12—C2—C3—C4	134.9 (4)	N2—C1—N1—C5	−176.2 (2)
C2—C3—C4—N1	−5.7 (6)	S1—C1—N1—C5	5.0 (3)
C2—C3—C4—C13	177.4 (4)	C3—C4—N1—C1	−8.0 (5)
C10—C5—C6—C7	1.2 (4)	C13—C4—N1—C1	169.1 (3)
N1—C5—C6—C7	−176.1 (2)	C3—C4—N1—C5	174.3 (3)
C5—C6—C7—C8	−1.4 (4)	C13—C4—N1—C5	−8.6 (4)
C5—C6—C7—Cl1	177.59 (19)	C6—C5—N1—C1	−90.2 (3)
C6—C7—C8—C9	0.5 (4)	C10—C5—N1—C1	92.5 (3)
Cl1—C7—C8—C9	−178.4 (2)	C6—C5—N1—C4	87.5 (3)
C6—C7—C8—Cl2	−179.8 (2)	C10—C5—N1—C4	−89.7 (3)
Cl1—C7—C8—Cl2	1.3 (3)	N1—C1—N2—C2	9.7 (4)
C7—C8—C9—C10	0.5 (4)	S1—C1—N2—C2	−171.5 (2)
Cl2—C8—C9—C10	−179.2 (2)	C3—C2—N2—C1	−20.4 (4)
C6—C5—C10—C9	−0.1 (4)	C11—C2—N2—C1	101.9 (4)
N1—C5—C10—C9	177.1 (3)	C12—C2—N2—C1	−139.4 (3)
C8—C9—C10—C5	−0.7 (5)		

### 1-(3,4-Dichlorophenyl)-4,4,6-trimethyl-3,4-dihydropyrimidine-2(1H)-thione Hydrogen-bond geometry (Å, º)

**Table:**

*D*—H···*A*	*D*—H	H···*A*	*D*···*A*	*D*—H···*A*
N2—H2*N*···S1^i^	0.85 (2)	2.55 (2)	3.375 (2)	163 (3)

Symmetry code: (i) −*x*, −*y*+1, −*z*.
