# Supplementary material for: Synthesis, crystal structure and Hirshfeld surface analysis of 1-(3,4-di­chloro­phen­yl)-4,4,6-trimethyl-3,4-di­hydro­pyrimidine-2(1H)-thione
Source: Acta Crystallogr E Crystallogr Commun. 2026 Jul 16;82(Pt 8):937–41. doi: 10.1107/S2056989026007231 (PMC13446471; doi:10.1107/S2056989026007231)

checkCIF/PLATON report

Structure factors have been supplied for datablock(s) shelx

THIS REPORT IS FOR GUIDANCE ONLY. IF USED AS PART OF A REVIEW PROCEDURE FOR PUBLICATION, IT SHOULD NOT REPLACE THE EXPERTISE OF AN EXPERIENCED CRYSTALLOGRAPHIC REFEREE.

No syntax errors found.   CIF dictionary   Interpreting this report

Datablock: shelx

```
Bond precision:   C-C = 0.0048 A          Wavelength=0.71073

Cell:            a=7.916(1)       b=8.927(1)        c=11.759(2)
                 alpha=99.93(1)   beta=107.18(1)    gamma=102.32(1)
Temperature:     293 K

                 Calculated                 Reported
Volume           750.5(2)                   750.52(19)
Space group      P -1                       P -1
Hall group       -P 1                       -P 1
Moiety formula   C13 H14 Cl2 N2 S           C13 H14 Cl2 N2 S
Sum formula      C13 H14 Cl2 N2 S           C13 H14 Cl2 N2 S
Mr               301.22                     301.22
Dx,g cm-3        1.333                      1.333
Z                2                          2
Mu (mm-1)        0.556                      0.556
F000             312.0                      312.0
F000'            312.91
h,k,lmax         9,11,14                    9,11,14
Nref             3071                       3026
Tmin,Tmax        0.786,0.828                0.769,0.834
Tmin'            0.757

Correction method= # Reported T Limits: Tmin=0.769 Tmax=0.834
AbsCorr = MULTI-SCAN
```

Data completeness= 0.985   Theta(max)= 26.370

R(reflections)= 0.0473(2502)   wR2(reflections)= 0.1314(3026)

S = 1.086   Npar= 166

The following ALERTS were generated. Each ALERT has the format
    test-name_ALERT_alert-type_alert-level.
Click on the hyperlinks for more details of the test.

Alert level C

```
PLAT241_ALERT_2_C High  MainResAtom  Ueq as Compared to Neighbours         C3 Check
PLAT242_ALERT_2_C Low   MainResAtom  Ueq as Compared to Neighbours         C2 Check
PLAT334_ALERT_2_C Small <C-C> Benzene Dist.   C5       -C10      .       1.37 Ang.
PLAT340_ALERT_3_C Low Bond Precision on  C-C Bonds ..............    0.00482 Ang.
PLAT906_ALERT_3_C Large K Value in the Analysis of Variance ......      3.046 Check
PLAT911_ALERT_3_C Missing FCF Refl Between Thmin & STh/L=    0.600         19 Report
                8 -7  1,  -1  1  1,   6 -9  2,   8  0  2,  -7  0  3,  -9  4  4,
                1  8  4,  -9  1  5,  -4  9  5,   5  2  6,  -1  7  6,   1 -7 11,
                2 -3 11,   0  0 11,  -4 -4 12,   1 -1 12,  -3 -5 13,   0 -1 13,
               -4  2 13,
```

Alert level G

```
PLAT002_ALERT_2_G Number of Distance or Angle Restraints on AtSite          2 Note
PLAT154_ALERT_1_G The s.u.'s on the Cell Angles are Equal ..(Note)       0.01 Degree
PLAT172_ALERT_4_G The CIF-Embedded .res File Contains DFIX Records          1 Report
PLAT199_ALERT_1_G Reported _cell_measurement_temperature ..... (K)        293 Check
PLAT200_ALERT_1_G Reported   _diffrn_ambient_temperature ..... (K)        293 Check
PLAT380_ALERT_4_G Incorrectly? Oriented X(sp2)-Methyl Moiety .....        C13 Check
PLAT860_ALERT_3_G Number of Least-Squares Restraints ............           1 Note
PLAT883_ALERT_1_G Absent Datum for _atom_sites_solution_primary ..     Please Do !
PLAT899_ALERT_4_G SHELXL2018 is Outdated   and Succeeded by SHELXL     2019/3 Note
PLAT910_ALERT_3_G Missing FCF Reflection(s) Below Theta(Min)[Deg]=       2.66 Note
                0  1  0,   0  0  1,
PLAT912_ALERT_4_G Missing # of FCF Reflections Above STh/L=  0.600         24 Note
PLAT941_ALERT_3_G Average HKL Measurement Multiplicity ..........         1.7 Low
PLAT969_ALERT_5_G The 'Henn et al.' R-Factor-gap value ..........      11.114 Note
              Predicted wR2: Based on SigI**2  1.18 or SHELX Weight 12.10 Note
PLAT978_ALERT_2_G Number C-C Bonds with Positive Residual Density.          0 Info
```

---

0 **ALERT level A** = Most likely a serious problem - resolve or explain
0 **ALERT level B** = A potentially serious problem, consider carefully
6 **ALERT level C** = Check. Ensure it is not caused by an omission or oversight
14 **ALERT level G** = General information/check it is not something unexpected

4 ALERT type 1 CIF construction/syntax error, inconsistent or missing data
5 ALERT type 2 Indicator that the structure model may be wrong or deficient
6 ALERT type 3 Indicator that the structure quality may be low
4 ALERT type 4 Improvement, methodology, query or suggestion
1 ALERT type 5 Informative message, check

Publication of your CIF

You should attempt to resolve as many as possible of the alerts in all categories. Often the minor alerts point to easily fixed oversights, errors and omissions in your CIF or refinement strategy, so attention to these fine details can be worthwhile. In order to resolve some of the more serious problems it may be necessary to carry out additional measurements or structure refinements. However, the nature of your study may justify the reported deviations from journal submission requirements and the more serious of these should be commented upon in the discussion or experimental section of a paper or in the "special_details" fields of the CIF. *checkCIF* was carefully designed to identify outliers and unusual parameters, but every test has its limitations and alerts that are not important in a particular case may appear. Conversely, the absence of alerts does not guarantee there are no aspects of the results needing attention. It is up to the individual to critically assess their own results and, if necessary, seek expert advice.

If you wish to submit your CIF for publication in Acta Crystallographica Section C or E, you should upload your CIF via the web. If you wish to submit your CIF for publication in IUCrData you should upload your CIF via the web. If your CIF is to form part of a submission to another IUCr journal, you will be asked, either during electronic submission or by the Co-editor handling your paper, to upload your CIF via our web site.

PLATON version of 23/04/2026; check.def file version of 30/03/2026

Datablock shelx - ellipsoid plot

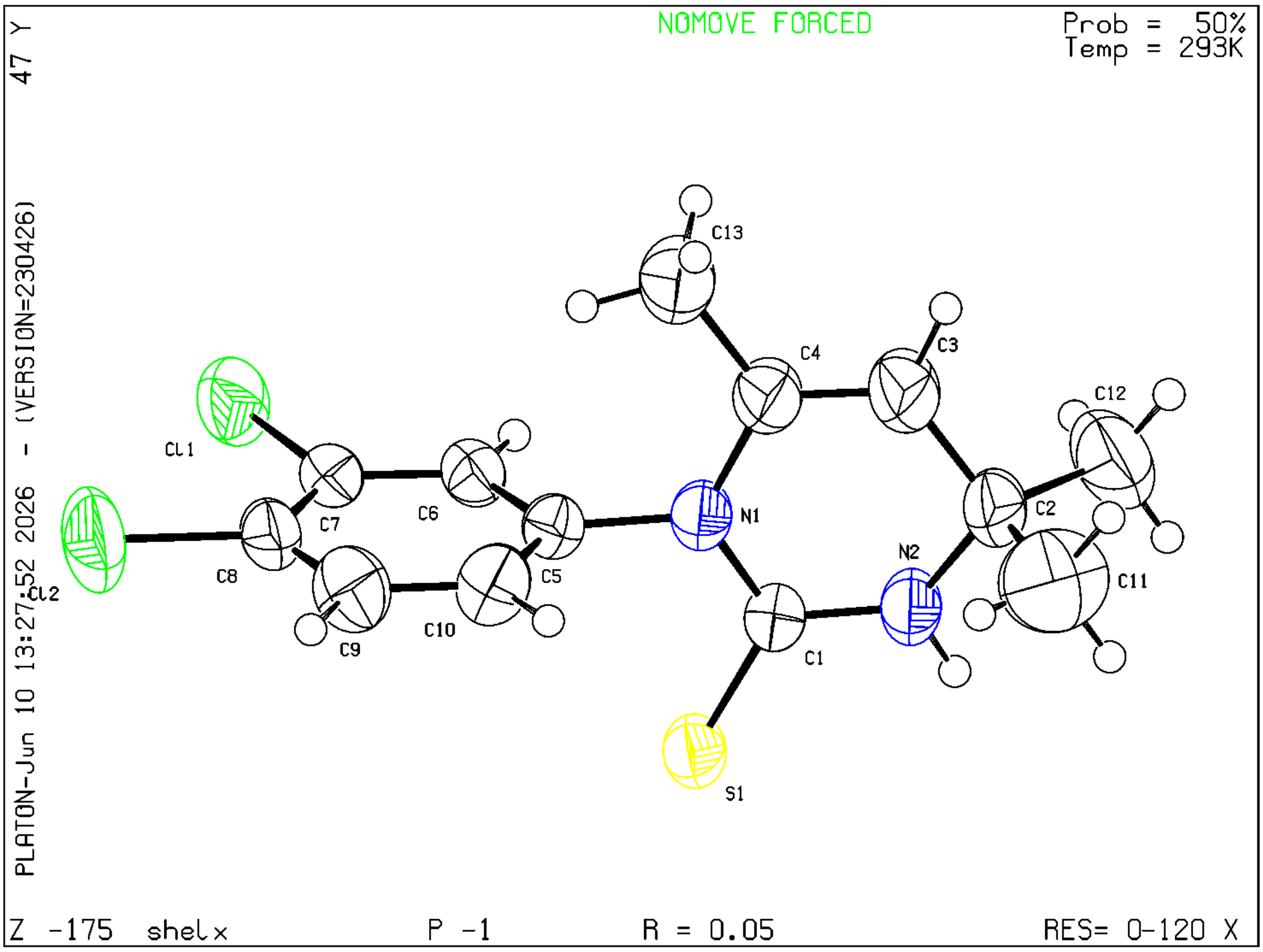

Supplement: Supplementary file 1 [file e-82-00937-sup3.pdf]
