# Supplementary material for: Synthesis, crystal structure and Hirshfeld surface analysis of 1-(3,4-di­chloro­phen­yl)-4,4,6-trimethyl-3,4-di­hydro­pyrimidine-2(1H)-thione
Source: Acta Crystallogr E Crystallogr Commun. 2026 Jul 16;82(Pt 8):937–41. doi: 10.1107/S2056989026007231 (PMC13446471; doi:10.1107/S2056989026007231)
Supplement: Supplementary file 4 [file e-82-00937-sup6.pdf]

Synthesis, crystal structure and Hirshfeld surface analyses of 1-(3,4-dichloro-phenyl)-4,4,6-trimethyl-3,4- dihydropyrimidine-2(1*H*)-thione

Sharatha Kumar[a]* and Tuncer Hökelek[b]

[a]Department of Chemistry, Yenepoya Institute of Arts, Science, Commerce and Management, Mangaluru, Yenepoya University (Deemed to be University), 575013 Karnataka, India, and [b]Hacettepe University, Department of Physics, 06800 Beytepe-Ankara, Türkiye
Correspondence email: sharathyiascm@yenepoya.edu.in

Abstract

The title compound, $C_{10}H_{11}ClN_2S$, consists of the chlorophenyl ring and propinoyl moiety bridged over the thiourea functional group. The dihedral angle between the planar propinoyl and thiourea groups is 8.33 (14)°, and they are orinted with respect to the phenyl ring at 48.50 (14)° and 56.09 (6)°. The intramolecular N—H···O hydrogen bond forms an S(6) ring motif. In the crystal, intermolecular N—H···S hydrogen bonds link the molecules, enclosing $R^2_2(8)$ ring motifs, into centrosymmetric dimers. Further, the $\pi\cdots\pi$ stacking interactions may help to consolidate the packing. Hirshfeld surface analysis revealed that the most important contributions for the crystal packing are from H ··· H (39.2%), H ··· Cl/Cl ··· H (15.8%), H ··· S/S ··· H (14.2%) and H ··· C/C ··· H (9.9%) interactions. The volume of the crystal voids and the percentage of free space were calculated to be 105.62 Å^3 and 9.33%, showing that there is no large cavity in the crystal packing. Computational methods revealed N—H···S hydrogen-bonding energy of −53.5 kJ mol^{-1}. Evaluations of the electrostatic, dispersion and total energy frameworks indicate that the stabilization is dominated *via* the electrostatic energy contributions.

1. Chemical context

Heterocyclic compounds containing pyrimidine and dihydropyrimidine frameworks have attracted sustained interest because of their wide range of biological and pharmacological activities, including antibacterial, antifungal, antitubercular and ant-inflammatory properties (Kappe, 2000; Leite *et al.*, 2026; Sriram, 2006). In particular, Biginelli-type dihydropyrimidine derivatives are recognized as important structural motifs in medicinal chemistry and synthetic organic chemistry owing to their diverse therapeutic potentials and convenient synthetic accessibilities. Continuing our interest in the syntheses and structural characterizations of biologically significant heterocyclic compounds (Alam *et al.*, 2005), the 1-(3,4- dichlorophenyl)-4,4,6-trimethyl-3,4-dihydropyrimidine-2(1*H*)- thione (I) was prepared and its molecular and crystal structures investigated by single-crystal X-ray diffraction analysis.

Herein, we reported the molecular and crystal structures together with Hirshfeld surface (HS) and crystal void analyses and interaction energy calculations and energy frameworks of the title compound, (I) (see Scheme I).

2. Structural commentary

The title compound, (I), contains the planar phenyl ring and the six-membered nitrogen containing heterocyclic dihydropyrimidine thione ring (Fig. 1). The 3,4-dichlorophenyl, dimethyl and methyl substituents are bonded to the 3,4-dihydropyrimidine-2(1*H*)-thione ring at N1, C2 and C4 positions, respectively. The pyrimidine, *A* (N1/N2/C1—C4), ring is in flattened-boat conformation (Fig. 2) with the puckering parameters (Cremer & Pople, 1975) of Q_T = 0.184 (3) Å, θ = 70.8 (9)° and φ = 174.4 (11)°. It is reported as planar in 4,4,6-trimethyl-1-phenyl-3,4-dihydro- pyrimidine-2(1*H*)-thione [(II); Yamin *et al.*, 2005], *N*-benzoyl-*N*′-phenyl-thiourea [(III); Yamin & Yusof, 2003] and 1-ethyl-5-(4-methoxybenzoyl)-4-(4-methoxyphenyl)pyrimidine-2(1*H*)- thione [(IV); Özçelik *et al.*, 2004]. The S1═C1 [1.692 (2) Å], C1—N1 [1.361 (3) Å] and C1—N2 [1.323 (3) Å] bond lengths are comparable with the correponding values in

compounds II—IV. Other bond lengths are in normal ranges (Allen *et al.*, 1987).

3. Supramolecular features

In the crystal, intermolecular N—H···S hydrogen bonds (Table 2) link the molecules, enclosing $R^2_2(8)$ ring motifs (Etter *et al.*, 1990), into centrosymmetric dimers (Fig. 3*a*). The supramolecular dimer formation through the intermolecular N—H···S hydrogen bonds is shown in Fig. 3*b*. Neither $\pi\cdots\pi$ stacking nor C—H···π(ring interactions are observed.

The intermolecular interactions in the crystal are visualized by carrying out the Hirshfeld surface (HS) analysis using *CrystalExplorer* 17.5 (Spackman *et al.*, 2021). Figure 4 shows the Hirshfeld surface with several neighboring molecules in the crystal. The white surface indicates contacts with distances equal to the sum of van der Waals radii, and the red and blue colours indicate distances shorter (in close contact) or longer (distinct contacts) than the van der Waals radii, respectively. The red spots indicate their roles as the respective donors and/or acceptors atoms in hydrogen bonding, as discussed; they also appear as the blue and red regions corresponding to positive and negative potentials on the HS mapped over electrostatic potential as shown in Fig. 5. The blue and red regions indicate positive (hydrogen-bond donors) and negative (hydrogen-bond acceptors) electrostatic potentials.

The overall two-dimensional fingerprint plot is shown in Fig. 6*a* and those delineated into H ··· H, H ··· Cl/Cl ··· H, H ··· S/S ··· H, H ··· C/C ··· H, C ··· Cl/Cl ··· C, Cl ··· Cl, N ··· Cl/Cl ··· N, H ··· N/N ··· H, C ··· C and S ··· Cl/Cl ··· S interactions are illustrated in Figs. 6 (*b*–*k*), respectively. According to the two-dimensional fingerprint plots the H ··· H, H ··· Cl/Cl ··· H, and H ··· Cl/Cl ··· H contacts make the most significant contributions to the HS, at 40.8%, 28.7% and 15.5%, respectively (Fig. 6).

The strength of the crystal packing depends on the tightly packing of the molecules which results insignificant voids. For checking the strength of the crystal, a void analysis was performed. The volume of the crystal voids (Figs. 7 *a* and *b*) and the percentage of free space in the unit cell calculated as 135.01 Å^3 and 17.99%, respectively. Thus, crystal packing appears not compact.

The intermolecular interaction energies are calculated using CE–B3LYP/6–31 G(d,p) energy model available in *CrystalExplorer* 17.5 (Spackman *et al.*, 2021), where a cluster of molecules is generated by applying crystallographic symmetry operations with respect to a selected central molecule within the radius of 3.8 Å by default. The total intermolecular energy (E_{tot}) is the sum of electrostatic (E_{ele}), polarization (E_{pol}), dispersion (E_{dis}) and exchange-repulsion (E_{rep}) energies (Turner *et al.*, 2015) with scale factors of 1.057, 0.740, 0.871 and 0.618, respectively (Mackenzie *et al.*, 2017). Hydrogen bonding interaction energies (in kJ mol^{-1}) were calculated to be −80.2 (E_{ele}), −20.0 (E_{pol}), −22.8 (E_{dis}), 70.4 (E_{rep}) and −58.2 (E_{tot}) for N2–H2N–S1 hydrogen bonding interactions.

Energy frameworks combine the calculation of intermolecular interaction energies with a graphical representation of their magnitudes, in which they were constructed for E_{ele} (red cylinders), E_{dis} (green cylinders) and E_{tot} (blue cylinders) (Figs. 8 *a*, *b* and *c*). The evaluations of the electrostatic, dispersion and total energy frameworks indicate that the stabilization is dominated *via* the electrostatic energy contributions in the crystal structure of the title compound.

4. Database survey

A search of the Cambridge Structural Database (CSD, Version 6.00, updated May 2025; Groom *et al.*, 2016) revealed six closely related structures of 1-aryl-4,4,6-trimethyl-3,4- dihydropyrimidine-2(1*H*)-thione derivatives. These include: 1-(4-fluorophenyl)-4,4,6-trimethyl-3,4-dihydropyrimidine- 2(1*H*)-thione, $C_{13}H_{15}FN_2S$ (CSD refcode ASEHIR; Kadir *et al.*, 2016), 1-(3-chlorophenyl)-4,4,6-trimethyl-3,4- dihydropyrimidine-2(1*H*)-thione, $C_{13}H_{15}ClN_2S$ (CSD refcode DN2640; Yamin & Salem, 2011*a*), 1-(3-fluorophenyl)-4,4, 6-trimethyl-3,4-dihydropyrimidine-2(1*H*)-thione, $C_{13}H_{15}FN_2S$ (CSD refcode LD2016; Yamin *et al.*, 2011*b*), 4,4,6-trimethyl-1-phenyl-3,4-dihydropyrimidine- 2(1*H*)-thione, $C_{13}H_{16}N_2S$

(Yamin *et al.*, 2005), 1-(4-chlorophenyl)-4,4,6-trimethyl-3,4-dihydropyrimidine- 2(1*H*)-thione, $C_{13}H_{15}ClN_2S$ (CSD refcode ZQ2028; Saeed & Bolte, 2010*a*) and 4,4,6-trimethyl-1-(3-methylphenyl)- 3,4-dihydropyrimidine-2(1*H*)-thione, $C_{14}H_{18}N_2S$ (CSD refcode ZS2028; Saeed *et al.*, 2010*b*). The title compound differs from these reproted structures by the presence of chlorine substituents at both the 3- and 4- positions of the phenyl ring. Similar to several related derivatives, the crystal packing is stabilized by intermolecular N—H···S hydrogen bonds, forming centrosymmetric dimers.

5. Synthesis and crystallization

Portion-wise 3,4-dichloro aniline (0.16 g, 1.0 mmol) was added to a stirred solution of potassium thiocyanate (0.09 g, 1.0 mmol) in acetone containing one drop of H_2SO_4 at room temperature. The reaction mixture was then heated at 50–60 °C for 3 h while being monitored using thin-layer chromatography. After the reaction was completed, the content was cooled to room temperature, and then poured into ice water. The resulting precipitate was filtered, dried and recrystallized at room temperature from ethanol by gradual evaporation. Colourless crystals suitable for X-ray analysis were obtained by slow evaporation of acetonitrile. Colourless, yield 86%, mp 195 °C, IR (KBr) cm^{-1}: 3285 (N—H str.), 1530 (C═S str.), 3176 (Ar. C—H str.).

6. Refinement

Crystal data, data collection and structure refinement details are summarized in Table 1. The NH hydrogen atoms are located from a difference Fourier map and refined isotropically. The C-bonded hydrogen atom positions were calculated geometrically at distances of 0.93 Å (for aromatic CH) and 0.96 Å (for methyl CH) and refined using a riding model by applying the constraint of U_{iso} = k × U_{eq} (C), where k = 1.5 for methyl H atoms and k = 1.2 for the other H atoms.

Table 1

Experimental details

Crystal data	
Chemical formula	$C_{13}H_{14}Cl_2N_2S$
M_r	301.22
Crystal system, space group	Triclinic, $P\overline{1}$
Temperature (K)	293
a, *b*, *c* (Å)	7.916 (1), 8.927 (1), 11.759 (2)
α, β, γ (°)	99.93 (1), 107.18 (1), 102.32 (1)
V (Å^3)	750.52 (19)
Z	2
Radiation type	Mo *K*α
μ (mm^{-1})	0.56
Crystal size (mm)	0.50 × 0.36 × 0.34
Data collection	
Diffractometer	Oxford Diffraction Xcalibur (TM) Single Crystal X-ray Diffractometer with Sapphire CCD Detector.
Absorption correction	Multi-scan<br>*CrysAlis RED*, Oxford Diffraction Ltd., 2009 Empirical absorption correction using spherical harmonics, implemented in SCALE3 ABSPACK scaling algorithm.
T_{min}, T_{max}	0.769, 0.834
No. of measured, independent and observed [$I > 2\sigma(I)$] reflections	5185, 3026, 2502

R_{int}	0.014
$(\sin \theta/\lambda)_{max}$ (Å^{-1})	0.625
Refinement	
$R[F^2 > 2\sigma(F^2)]$, $wR(F^2)$, S	0.047, 0.131, 1.09
No. of reflections	3026
No. of parameters	166
No. of restraints	1
H-atom treatment	H atoms treated by a mixture of independent and constrained refinement
$\Delta\rho_{max}$, $\Delta\rho_{min}$ (e Å^{-3})	0.34, −0.36

Computer programs: *CrysAlis CCD* (Oxford Diffraction Ltd., 2006), *CrysAlis RED* (Oxford Diffraction Ltd., 2006), *SHELXT2014*/5 (Sheldrick, 2015a), *SHELXL2018*/3 (Sheldrick, 2015b), *ORTEP-3* for Windows (Farrugia, 2012), *WinGX* publication routines (Farrugia, 2012) and *PLATON* (Spek, 2009).

Table 2

Hydrogen-bond geometry (Å, º)

D—H···*A*	*D*—H	H···*A*	*D*···*A*	*D*—H···*A*
N2—H2*N*···S1^{i}	0.85 (2)	2.55 (2)	3.375 (2)	163 (3)

Symmetry code: (i) −*x*, −*y*+1, −*z*.

Acknowledgements

The authors thank the Yenepoya Deemed to be University for the facilities and financial support. T. H. is also grateful to Hacettepe University Scientific Research Project Unit (Grant No. 013 D04 602 004). The author's contributions are as follows. Conceptualization, SK and TH; synthesis, SK; X-ray analysis, SK and TH; Hirshfeld surface analysis, TH; writing (review and editing of the manuscript) SK and TH; supervision, TH and SK.

Funding information

References

Alam, O., Imran, M. & Khan, S. A. (2005). *Indian J. Heterocycl. Chem.* **14**, 293–296.

Allen, F. H., Kennard, O., Watson, D. G., Brammer, L., Orpen, A. G. & Taylor, R. (1987). *J. Chem. Soc. Perkin Trans.* **2**, S1–19.

Cremer, D. & Pople, J. A. (1975). *J. Am. Chem. Soc.* **97**, 1354–1358.

Etter, M. C., MacDonald, J. C. & Bernstein, J. (1990). *Acta Cryst.* E**46**, 256–262.

Farrugia, L. J. (2012). *J. Appl. Cryst.* **45**, 849–854.

Groom, C. R., Bruno, I. J., Lightfoot, M. P. & Ward, S. C. (2016). *Acta Cryst.* B**72**, 171–179.

Kadir, A., Abd Malek, N. A., Mohd Zaki, H., Hasbullah, S. A. & Yamin, B. M. (2016). *IUCrData* **1**, x161189.

Kappe, C. O. (2000). *Acc. Chem. Res.* **33**, 879–888.

Leite, A. C. L., Lima, R. S., Moreira, D. R. M., Cardoso, M. V. O., Brito, A. C. G., Santos, L. M. F., Hernandes, M. Z., Kiperstok, A. C., Lima, R. S. & Soares, M. B. P. (2006). *Bioorg. Med. Chem.* **14**, 3749–3757.

Mackenzie, C. F., Spackman, P. R., Jayatilaka, D. & Spackman, M. A. (2017). *IUCrJ* **4**, 575–587.

Oxford Diffraction (2006). *CrysAlis CCD* and *CrysAlis RED* Oxford Diffraction Ltd, Abingdon, Oxfordshire, England.

Özçelik, S., Dinçer, M., Yildirim, I. & Akçamur, Y. (2004). *Acta Cryst.* E**60**, o1123–o1125.

Saeed, A. & Bolte, M. (2010*a*). *Acta Cryst.* E**66**, o440.

Saeed, A., Khera, R. A. & Parvez, M. (2010*b*). *Acta Cryst.* E**66**, o635.

Sheldrick, G. M. (2015*a*). *Acta Cryst.* A**71**, 3–8.

Sheldrick, G. M. (2015*b*). *Acta Cryst.* C**71**, 3–8.

Spackman, P. R., Turner, M. J., McKinnon, J. J., Wolff, S. K., Grimwood, D. J., Jayatilaka, D. & Spackman, M. A. (2021). *J. Appl. Cryst.* **54**, 1006–1011.

Spek, A. L. (2009). *Acta Cryst.* D**65**, 148–155.

Sriram, D., Yogeeswari, P. & Devakaram, R. V. (2006). *Bioorg. Med. Chem.* **14**, 3113–3118.

Turner, M. J., Thomas, S. P., Shi, M. W., Jayatilaka, D. & Spackman, M. A. (2015). *Chem. Commun.* **51**, 3735–3738.

Yamin, B. M., Kasim, N. A. M. & Hamzah, N. (2005). *Acta Cryst.* E**61**, o55–o57.

Yamin, B. M., Lawi, R. L. & Salem, H. F. (2011*b*). *Acta Cryst.* E**67**, o1810.

Yamin, B. M. & Salem, H. F. (2011*a*). *Acta Cryst.* E**67**, o282.

Yamin, B. M. & Yusof, M. S. (2003). *Acta Cryst.* E**59**, o151–o152.

Figure 1

The asymmetric unit with atom-numbering scheme and 50% probability ellipsoids.

Figure 2

Conformation of the pyrimidine ring.

Figure 3

(*a*) The dimer formation through the intermolecular N—H···S hydrogen bonds shown as dashed lines with the $R^2_2(8)$ ring motifs, (*b*) A partial packing diagram viewed down the *b*-axis direction showing the supramolecular dimer formation. The intermolecular N—H···S hydrogen bonds are shown as dashed lines.

Figure 4

View of the three-dimensional Hirshfeld surface for molecule plotted over d_{norm} in the range from −0.3452 to 1.4957 a.u.

Figure 5

View of the three-dimensional Hirshfeld surface of the title compound plotted over electrostatic potential in the range of −0.0500 to 0.0500 a.u. using the STO-3 G basis set at the Hartree-Fock level of theory. Hydrogen-bond donors and acceptors are shown as blue and red regions around the atoms, corresponding to positive and negative potentials, respectively.

Figure 6

The full two-dimensional fingerprint plots for title molecule, showing (*a*) all interactions, and delineated into (*b*) H ··· H, (*c*) H ··· Cl/Cl ··· H, (*d*) H ··· S/S ··· H, (*e*) H ··· C/C ··· H, (*f*) C ··· Cl/Cl ··· C, (*g*) Cl ··· Cl, (*h*) N ··· Cl/Cl ··· N, (i) H ··· N/N ··· H, (*j*) C ··· C and (*k*) S ··· Cl/Cl ··· S interactions. The d_i and d_e values are the closest internal and external distances (in Å) from given points on the Hirshfeld surface contacts.

Figure 7

Crystal voids viewed down the (*a*) *a*-axis and (*b*) *b*-axis directions.

Figure 8

The energy frameworks for a cluster of molecules of the title compound viewed down the *a*-axis showing the (*a*) electrostatic energy, (*b*) dispersion energy and (*c*) total energy diagrams. The cylindirical radius is proportional to the relative strength of the corresponding energies and they were adjusted to the same scale factor of 80 with cut-off value of 5 kJ mol^{-1} within 2 *X* 2 *X* 2 unit cells.

supporting information

Synthesis, crystal structure and Hirshfeld surface analyses of 1-(3,4-dichlorophenyl)-4,4,6-trimethyl-3,4- dihydropyrimidine-2(1*H*)-thione

Computing details

Data collection: *CrysAlis CCD* (Oxford Diffraction Ltd., 2006); cell refinement: *CrysAlis RED* (Oxford Diffraction Ltd., 2006); data reduction: *CrysAlis RED* (Oxford Diffraction Ltd., 2006); program(s) used to solve structure: *SHELXT2014*/5 (Sheldrick, 2015a); program(s) used to refine structure: *SHELXL2018*/3 (Sheldrick, 2015b); molecular graphics: *ORTEP-3* for Windows (Farrugia, 2012); software used to prepare material for publication: *WinGX* publication routines (Farrugia, 2012) and *PLATON* (Spek, 2009).

(shelx)

Crystal data

$C_{13}H_{14}Cl_2N_2S$
M_r = 301.22
Triclinic, $P\overline{1}$
a = 7.916 (1) Å
b = 8.927 (1) Å
c = 11.759 (2) Å
α = 99.93 (1)°
β = 107.18 (1)°
γ = 102.32 (1)°
V = 750.52 (19) Å^3
Z = 2
F(000) = 312
D_x = 1.333 Mg m^{-3}
Mo $K\alpha$ radiation, λ = 0.71073 Å
Cell parameters from 2526 reflections
θ = 2.7–27.9°
μ = 0.56 mm^{-1}
T = 293 K
Prism, colourless
0.50 × 0.36 × 0.34 mm

Data collection

Oxford Diffraction Xcalibur (TM) Single Crystal X-ray Diffractometer with Sapphire CCD Detector.
Rotation method data acquisition using ω scans.
Absorption correction: multi-scan
CrysAlis RED, Oxford Diffraction Ltd., 2009 Empirical absorption correction using spherical harmonics, implemented in SCALE3 ABSPACK scaling algorithm.
T_{min} = 0.769, T_{max} = 0.834
5185 measured reflections
3026 independent reflections
2502 reflections with $I > 2\sigma(I)$
R_{int} = 0.014
θ_{max} = 26.4°, θ_{min} = 2.7°
h = −9→9
k = −10→11
l = −14→14

Refinement

Refinement on F^2
Least-squares matrix: full
$R[F^2 > 2\sigma(F^2)]$ = 0.047
$wR(F^2)$ = 0.131
S = 1.09
3026 reflections
166 parameters
1 restraint
Hydrogen site location: mixed
H atoms treated by a mixture of independent and constrained refinement
$w = 1/[\sigma^2(F_o^2) + (0.0531P)^2 + 0.5196P]$ where $P = (F_o^2 + 2F_c^2)/3$
$(\Delta/\sigma)_{max}$ < 0.001
$\Delta\rho_{max}$ = 0.34 e Å^{-3}
$\Delta\rho_{min}$ = −0.36 e Å^{-3}

Special details

Geometry. All e.s.d.'s (except the e.s.d. in the dihedral angle between two l.s. planes) are estimated using the full covariance matrix. The cell e.s.d.'s are taken into account individually in the estimation of e.s.d.'s in distances, angles and torsion angles; correlations between e.s.d.'s in cell parameters are only used when they are defined by crystal symmetry. An approximate (isotropic) treatment of cell e.s.d.'s is used for estimating e.s.d.'s involving l.s. planes.

Fractional atomic coordinates and isotropic or equivalent isotropic displacement parameters (Å²)

	x	*y*	*z*	U_{iso}*/U_{eq}
C1	0.3026 (3)	0.5937 (3)	0.1548 (2)	0.0422 (5)
C2	0.2695 (4)	0.8680 (3)	0.1621 (3)	0.0603 (7)
C3	0.4444 (5)	0.9152 (4)	0.2678 (4)	0.0816 (11)
H3	0.485846	1.019411	0.314955	0.098*
C4	0.5456 (4)	0.8184 (3)	0.2995 (3)	0.0627 (8)
C5	0.5946 (3)	0.5528 (3)	0.2628 (2)	0.0434 (5)
C6	0.5958 (3)	0.4814 (3)	0.3573 (2)	0.0465 (6)
H6	0.516423	0.494028	0.400471	0.056*
C7	0.7150 (4)	0.3909 (3)	0.3879 (2)	0.0480 (6)
C8	0.8296 (4)	0.3696 (3)	0.3222 (3)	0.0540 (6)
C9	0.8260 (4)	0.4402 (4)	0.2263 (3)	0.0649 (8)
H9	0.902697	0.425268	0.181383	0.078*
C10	0.7088 (4)	0.5327 (4)	0.1971 (3)	0.0574 (7)
H10	0.707063	0.581304	0.133010	0.069*
C11	0.2915 (7)	0.9217 (5)	0.0513 (4)	0.1110 (16)
H11A	0.333986	1.035250	0.071888	0.166*
H11B	0.379489	0.878031	0.026262	0.166*
H11C	0.174966	0.885896	−0.014726	0.166*
C12	0.1231 (6)	0.9308 (5)	0.1992 (5)	0.1088 (15)
H12A	0.162291	1.044564	0.221850	0.163*
H12B	0.008837	0.894392	0.131086	0.163*
H12C	0.106251	0.892958	0.267781	0.163*
C13	0.7245 (5)	0.8681 (4)	0.4024 (4)	0.0966 (14)
H13A	0.773023	0.778897	0.408496	0.145*
H13B	0.809630	0.949830	0.387448	0.145*
H13C	0.706552	0.907897	0.477922	0.145*
N1	0.4778 (3)	0.6555 (2)	0.23637 (19)	0.0465 (5)
N2	0.2043 (3)	0.6938 (3)	0.1281 (2)	0.0554 (6)
Cl1	0.71964 (14)	0.30829 (10)	0.51126 (7)	0.0761 (3)
Cl2	0.98030 (13)	0.25664 (12)	0.35758 (10)	0.0907 (3)
S1	0.21347 (9)	0.39621 (7)	0.09242 (7)	0.0518 (2)
H2N	0.094 (3)	0.652 (3)	0.078 (2)	0.062*

Atomic displacement parameters (Å²)

	U^{11}	U^{22}	U^{33}	U^{12}	U^{13}	U^{23}
C1	0.0414 (12)	0.0430 (12)	0.0441 (12)	0.0150 (10)	0.0137 (10)	0.0139 (10)
C2	0.0642 (17)	0.0437 (14)	0.0679 (18)	0.0224 (13)	0.0089 (14)	0.0166 (13)
C3	0.079 (2)	0.0437 (16)	0.095 (3)	0.0202 (15)	−0.0047 (19)	0.0087 (16)
C4	0.0581 (16)	0.0415 (14)	0.0708 (19)	0.0116 (12)	0.0006 (14)	0.0107 (13)
C5	0.0388 (12)	0.0433 (12)	0.0455 (13)	0.0140 (10)	0.0106 (10)	0.0082 (10)
C6	0.0527 (14)	0.0453 (13)	0.0455 (13)	0.0204 (11)	0.0185 (11)	0.0102 (10)
C7	0.0562 (15)	0.0394 (12)	0.0436 (13)	0.0176 (11)	0.0097 (11)	0.0068 (10)

C8	0.0468 (14)	0.0476 (14)	0.0623 (16)	0.0227 (11)	0.0094 (12)	0.0052 (12)
C9	0.0557 (17)	0.078 (2)	0.0727 (19)	0.0291 (15)	0.0333 (15)	0.0167 (16)
C10	0.0533 (15)	0.0718 (18)	0.0558 (16)	0.0226 (13)	0.0234 (13)	0.0240 (14)
C11	0.144 (4)	0.072 (2)	0.103 (3)	0.007 (3)	0.028 (3)	0.042 (2)
C12	0.090 (3)	0.083 (3)	0.138 (4)	0.046 (2)	0.020 (3)	−0.007 (3)
C13	0.075 (2)	0.0499 (17)	0.118 (3)	0.0162 (16)	−0.022 (2)	0.0000 (18)
N1	0.0443 (11)	0.0431 (11)	0.0505 (12)	0.0162 (9)	0.0104 (9)	0.0134 (9)
N2	0.0468 (12)	0.0428 (12)	0.0681 (15)	0.0166 (10)	0.0048 (11)	0.0137 (10)
Cl1	0.1138 (7)	0.0656 (5)	0.0650 (5)	0.0473 (5)	0.0301 (5)	0.0312 (4)
Cl2	0.0794 (6)	0.0843 (6)	0.1211 (8)	0.0566 (5)	0.0286 (5)	0.0269 (5)
S1	0.0471 (4)	0.0416 (3)	0.0619 (4)	0.0150 (3)	0.0103 (3)	0.0129 (3)

Geometric parameters (Å, º)

C1—N2	1.323 (3)	C7—Cl1	1.732 (3)
C1—N1	1.361 (3)	C8—C9	1.379 (4)
C1—S1	1.692 (2)	C8—Cl2	1.726 (3)
C2—N2	1.472 (3)	C9—C10	1.378 (4)
C2—C3	1.480 (4)	C9—H9	0.9300
C2—C11	1.507 (5)	C10—H10	0.9300
C2—C12	1.529 (5)	C11—H11A	0.9600
C3—C4	1.326 (4)	C11—H11B	0.9600
C3—H3	0.9300	C11—H11C	0.9600
C4—N1	1.419 (3)	C12—H12A	0.9600
C4—C13	1.484 (4)	C12—H12B	0.9600
C5—C6	1.371 (3)	C12—H12C	0.9600
C5—C10	1.372 (4)	C13—H13A	0.9600
C5—N1	1.443 (3)	C13—H13B	0.9600
C6—C7	1.378 (3)	C13—H13C	0.9600
C6—H6	0.9300	N2—H2N	0.851 (17)
C7—C8	1.376 (4)		
N2—C1—N1	117.4 (2)	C8—C9—H9	120.0
N2—C1—S1	121.53 (19)	C5—C10—C9	119.7 (3)
N1—C1—S1	121.06 (17)	C5—C10—H10	120.1
N2—C2—C3	107.2 (2)	C9—C10—H10	120.1
N2—C2—C11	108.3 (3)	C2—C11—H11A	109.5
C3—C2—C11	113.0 (3)	C2—C11—H11B	109.5
N2—C2—C12	107.8 (3)	H11A—C11—H11B	109.5
C3—C2—C12	110.5 (3)	C2—C11—H11C	109.5
C11—C2—C12	109.8 (3)	H11A—C11—H11C	109.5
C4—C3—C2	124.2 (3)	H11B—C11—H11C	109.5
C4—C3—H3	117.9	C2—C12—H12A	109.5
C2—C3—H3	117.9	C2—C12—H12B	109.5
C3—C4—N1	119.2 (3)	H12A—C12—H12B	109.5
C3—C4—C13	123.9 (3)	C2—C12—H12C	109.5
N1—C4—C13	116.9 (2)	H12A—C12—H12C	109.5
C6—C5—C10	120.6 (2)	H12B—C12—H12C	109.5
C6—C5—N1	119.4 (2)	C4—C13—H13A	109.5
C10—C5—N1	119.9 (2)	C4—C13—H13B	109.5
C5—C6—C7	119.8 (2)	H13A—C13—H13B	109.5
C5—C6—H6	120.1	C4—C13—H13C	109.5

C7—C6—H6	120.1	H13A—C13—H13C	109.5
C8—C7—C6	120.0 (2)	H13B—C13—H13C	109.5
C8—C7—Cl1	121.2 (2)	C1—N1—C4	121.1 (2)
C6—C7—Cl1	118.7 (2)	C1—N1—C5	119.6 (2)
C7—C8—C9	119.9 (2)	C4—N1—C5	119.2 (2)
C7—C8—Cl2	121.4 (2)	C1—N2—C2	127.5 (2)
C9—C8—Cl2	118.8 (2)	C1—N2—H2N	116 (2)
C10—C9—C8	120.0 (3)	C2—N2—H2N	116 (2)
C10—C9—H9	120.0		

N2—C2—C3—C4	17.7 (5)	N2—C1—N1—C4	6.2 (4)
C11—C2—C3—C4	−101.6 (5)	S1—C1—N1—C4	−172.6 (2)
C12—C2—C3—C4	134.9 (4)	N2—C1—N1—C5	−176.2 (2)
C2—C3—C4—N1	−5.7 (6)	S1—C1—N1—C5	5.0 (3)
C2—C3—C4—C13	177.4 (4)	C3—C4—N1—C1	−8.0 (5)
C10—C5—C6—C7	1.2 (4)	C13—C4—N1—C1	169.1 (3)
N1—C5—C6—C7	−176.1 (2)	C3—C4—N1—C5	174.3 (3)
C5—C6—C7—C8	−1.4 (4)	C13—C4—N1—C5	−8.6 (4)
C5—C6—C7—Cl1	177.59 (19)	C6—C5—N1—C1	−90.2 (3)
C6—C7—C8—C9	0.5 (4)	C10—C5—N1—C1	92.5 (3)
Cl1—C7—C8—C9	−178.4 (2)	C6—C5—N1—C4	87.5 (3)
C6—C7—C8—Cl2	−179.8 (2)	C10—C5—N1—C4	−89.7 (3)
Cl1—C7—C8—Cl2	1.3 (3)	N1—C1—N2—C2	9.7 (4)
C7—C8—C9—C10	0.5 (4)	S1—C1—N2—C2	−171.5 (2)
Cl2—C8—C9—C10	−179.2 (2)	C3—C2—N2—C1	−20.4 (4)
C6—C5—C10—C9	−0.1 (4)	C11—C2—N2—C1	101.9 (4)
N1—C5—C10—C9	177.1 (3)	C12—C2—N2—C1	−139.4 (3)
C8—C9—C10—C5	−0.7 (5)		

Hydrogen-bond geometry (Å, º)

D—H···*A*	*D*—H	H···*A*	*D*···*A*	*D*—H···*A*
N2—H2*N*···S1^{i}	0.85 (2)	2.55 (2)	3.375 (2)	163 (3)

Symmetry code: (i) −*x*, −*y*+1, −*z*.
